# Activity based costing system of continuous ambulatory peritoneal dialysis under the Universal Coverage Scheme in Thailand

**DOI:** 10.1186/1471-2458-14-S1-P7

**Published:** 2014-01-29

**Authors:** Boonruksa Laonapaporn, Pudtan Phanthunane

**Affiliations:** 1 Faculty of Medicine, Naresuan University, Phitsanulok 65000, Thailand

## Background

Thailand has introduced peritoneal dialysis (PD) as part of the Universal Health Coverage (UC) policy since 2008. However, its real cost based on patients’ background has not been revealed. The aim of this study was to analyse the total cost of continuous ambulatory peritoneal dialysis (CAPD) in Nopparat Rajathanee Hospital, Thailand.

## Materials and methods

The cost of CAPD per patient was computed using activity based costing (ABC) approach. We collected costs and outcomes data during the period from 1 January to 31 December 2011. One-way sensitivity analysis was adopted to evaluate the effect of factors that changes CAPD cost estimated. These factors included the difference in the hours physicians were available for clinical time; and drugs cost used in patients having diabetes mellitus (DM) and in non-DM patients. In addition to total patients, the survived and compliant patients were taken into account as the study’s outcomes.

## Results

Cost of CAPD service was estimated to be 57,929 Baht (USD1,931) per patient per year. It composed of materials and drugs cost of 43,519 Baht (77%), labour cost of 12,498 Baht (22%) and capital cost of 512 Baht (1%). We found that the difference in drug cost between patients with and without DM was only 102 Baht per patient per year. When changing outcome from total patients to survived patients (75%), the unit cost increased to 75,462 Baht. When only compliant patients (80%) were taken into account, the cost was estimated to be 70,746 Baht.

## Conclusions

The current study found that the cost of CAPD was higher than the reimbursement rate provided by the National Health Security Office (NHSO) of 48,000 Baht per patient per year.

